# Optimising Fully rPET-Sourced Aerogel Production Using a Sustainable Dissolution–Precipitation Approach

**DOI:** 10.3390/gels12060521

**Published:** 2026-06-10

**Authors:** Cláudio M. R. Almeida, David Gonçalves, Brigite Jorge, Pedro C. F. Silva, Tiago Cardoso, Pedro Nuno Simões, Ana C. Fonseca, Luisa Durães

**Affiliations:** 1 University of Coimbra, CERES, Department of Chemical Engineering, 3030-790 Coimbra, Portugalcesar@eq.uc.pt (P.C.F.S.); tiago.fernandes.cardoso@gmail.com (T.C.); pnsim@eq.uc.pt (P.N.S.); 2 University of Coimbra, CEMMPRE, ARISE, Department of Chemical Engineering, 3030-790 Coimbra, Portugal

**Keywords:** polymer waste upcycling, rPET dissolution, polymer aerogels, thermal insulation

## Abstract

Aerogels were produced exclusively from recycled plastic bottles of poly(ethylene terephthalate) (rPET) by optimising a dissolution–precipitation process at room temperature and applying a product design strategy to improve their sustainability. Using a design of experiments methodology, the systematic assessment of the influence of different factors, namely rPET concentration, co-solvent ratio, and non-solvent quantity, on the key properties of rPET aerogel, namely bulk density, thermal conductivity, and mechanical resistance, was performed. The understanding of the significance of each parameter and the optimisation of a desirability function offered reliable optimum results for the adjustment of the experimental procedure for the reduction in the volume of the most critical solvent, trifluoroacetic acid (TFA), by 42.5%. The observed bulk density values were excellent, down to 110 kg·m^−3^, and the thermal conductivity was in the range of conventional commercial insulators (38 mW·m^−1^·K^−1^), positioning this material as a real alternative to conventional thermal insulators. Also, to deeply understand the dissolution/precipitation phenomena, molecular dynamics simulations were conducted to support the experimental outcomes.

## 1. Introduction

The rapid growth in global plastic production has created severe environmental and waste-management challenges, particularly because of the persistence and low degradability of commodity polymers such as poly(ethylene terephthalate) (PET). PET is widely used in beverage bottles, food packaging, and textile fibres, and consequently represents a major fraction of post-consumer plastic waste streams. For example, over half a trillion PET bottles are discarded annually [1]. Incineration of PET to recover energy contributes to greenhouse gas emissions and pollution. A conventional PET recycling method is mechanical reprocessing, in which the collected waste is washed, shredded, and remelted, and often blended with virgin polymer. However, this method is limited by the thermal and hydrolytic degradation of polymer chains, which causes a decrease in the molecular weight and deterioration of the mechanical properties over repeated cycles [2]. Another recycling strategy is chemical recycling [2,3], a process that breaks the polymer down into its original building blocks or into oligomers, allowing it to be remade into new plastic or other products. Also, in this case, the high temperature and pressure required for the depolymerisation process and high costs compared to the mechanical recycling make this method not favourable for large-scale application.

As an alternative approach, solvent-based methods, which rely on the dissolution and subsequent precipitation of PET, have emerged as a promising strategy for recovering polymer chains while preserving the molecular structure and saving energy [4]. Thus, the development of recycling and upcycling strategies to recover value from these wastes is now under focus. In this context, transforming recycled PET (rPET) into high-value porous materials, such as aerogels, offers an attractive route for integrating polymer waste into a circular economy [5,6]. Their combination of performance, cost-effectiveness, and environmental benefits makes them attractive for insulation, remediation, acoustic damping, and advanced functional materials.

Solvent-based approaches exploit tailored solvent systems that can effectively disrupt intermolecular interactions in PET while maintaining chain integrity, enabling recovery via controlled phase separation (precipitation). For PET, mixed solvent systems containing strong hydrogen-bonding or acidic components combined with volatile co-solvents have been investigated to overcome its poor solubility under ambient conditions. In particular, mixtures of trifluoroacetic acid (TFA) and dichloromethane (DCM) have been reported to efficiently dissolve PET at room temperature [6]. In this case, TFA acts as a highly polar, protonating solvent for the ester carbonyl groups, and DCM reduces the solution viscosity and facilitates chain mobility. Thermodynamic studies on PET/[TFA + DCM]/water systems confirmed that such ternary systems exhibit well-defined cloud points and are suitable for phase-inversion processes, where the addition of a non-solvent (water) induces controlled phase separation and pore three-dimensional network formation [4].

Aerogels are ultralight materials characterised by porosities higher than 90% and a three-dimensional interconnected porous network, mostly formed by mesopores. These properties make them suitable for thermal and acoustic insulation. In a previous work [6], we developed a simple and straightforward method for the obtainment of rPET aerogels. The method distinguishes itself from the others published [7,8,9,10] because it does not use temperature for dissolution or crosslinkers to stabilise the structure of the aerogel.

rPET aerogels produced through a room-temperature dissolution/precipitation process represent a promising class of porous materials for environmental applications. Their highly porous structure, low density, and large specific surface area make them well-suite to address several environmental challenges while benefiting from an energy-efficient fabrication route. One of the most promising applications of PET aerogels is in water treatment and remediation [11]. The interconnected porous network of the aerogel can facilitate the adsorption of organic pollutants, dyes, and pharmaceuticals from contaminated water. rPET aerogels also show strong potential for oil spill cleanup and oil–water separation. Another important environmental application is in air purification and gas capture [12]. The porous architecture of rPET aerogels can be exploited for the removal of particulate matter and volatile organic compounds (VOCs) from air streams.

In this work, we aim to further optimise the preparation process mainly in terms of the quantity of the most critical solvent used (TFA). Thus, a dissolution/precipitation route employing a DCM/TFA solvent mixture and ethanol as a non-solvent was optimised to produce rPET aerogels with low bulk densities and thermal conductivities. By systematically studying the effects of formulation parameters and process conditions, such as the solvent ratio, polymer concentration, and ethanol addition, on the structural, mechanical and insulation properties of the resultant products, and combining this information with insights from molecular dynamics simulations, it was possible to establish for the first time processing–structure–property relationships for these recently developed materials. The performed optimisation was guided by sustainability principles, favouring the use of less hazardous chemicals. At the end, the optimal preparation parameters were established to produce rPET aerogels with optimised properties while reducing the use of environmentally harmful reagents.

## 2. Results and Discussion

### 2.1. DoE and Parameters’ Influence on Sample Properties

**Figure 1:**
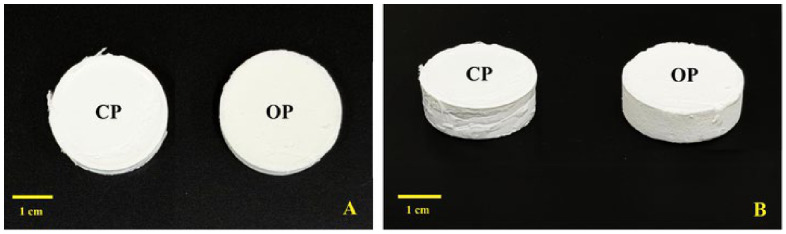
Visual aspect of rPET-based aerogels: (**A**) top and (**B**) side views of CP and OP samples.

The prepared samples are shown in Figure 1, and the selected properties are summarised in Table 1.

In all cases, the prepared aerogels exhibited low volume shrinkages, below 10%, suggesting that the samples preserved the highly porous network of the original gels. Low shrinkage values have been reported for rPET-based aerogels [6]. The desirability function method has proven to be useful for aerogel sample optimisation [13,14]. The same methodology was applied here to obtain the minimum values for the bulk density and thermal conductivity while maximising Young’s modulus. Under these conditions, the optimal preparation conditions were found to be rPET = 0.785 g, TFA = 2 mL, and EtOH = 1.2 mL. However, because one of the main goals was to reduce the use of the most environmental critical solvent, a sub-optimisation problem was defined by eliminating the TFA factor, and a second optimisation step was performed using the same responses and factor parametrisation. In this case, an optimum point was obtained with the following parameters: rPET = 0.7 g, TFA = 1.15 mL (minimum amount possible for polymer dissolution), and EtOH = 1.2 mL, corresponding to the calculated material property values of *ρ*_b_ = 0.111 g·cm^−3^, *k* = 38.4 mW·m^−1^·K^−1,^ and *Y*_M_ = 430 kPa. This was the used optimal point (OP) sample (equivalent to sample S2 in Table 1) for further characterisation, along with the central point (CP) sample for comparison. It is worth noting that the calculated properties are in very good agreement with those experimentally obtained.

rPET aerogels were produced via a dissolution/precipitation route, in which network formation is governed by thermodynamic processes [15]. In this approach, factors such as the polymer content, co-solvent, and non-solvent amounts determine the final pore architecture after the solvent exchange and drying steps. Consequently, properties such as bulk density, thermal conductivity, and Young’s modulus are primarily affected by the polymer skeleton, with significant contributions from the pore size distribution and interconnectivity.

The polymer content was the dominant factor controlling both the density and mechanical features. Increasing the rPET from 0.7 g (S1 to S4) to 1.4 g (S5 to S8) resulted in a clear shift toward denser and mechanically stronger aerogels. The samples with 0.7 g rPET exhibited low *ρ*_b_ in the range of 0.108 to 0.151 g·cm^−3^, with *k* values of 39.2–40.7 mW·m^−1^·K^−1^ and *Y*_M_ values from 243 to 524 kPa. In contrast, the 1.4 g samples showed substantially higher densities (0.230–0.274 g·cm^−3^), which is the reason for the higher *k* values (42.5–50.5 mW·m^−1^·K^−1^) and increased stiffness (1222–2139 kPa). The increase in *k* with the polymer content indicates that solid-phase conduction becomes more significant as the polymer network densifies and provides more continuous heat-transfer pathways.

#### 2.1.1. Influence of the Solvent System

Different behaviours were observed for the TFA content. For the 0.7 g series at EtOH = 0.8 mL, increasing TFA reduced *ρ*_b_ from 0.151 to 0.110 g·cm^−3^ (S1 vs. S3), while *k* presented similar values (40.7 vs. 39.4 mW·m^−1^·K^−1^). At EtOH = 1.2 mL, increasing TFA slightly increased *ρ*_b_ (0.108 to 0.119 g·cm^−3^; S2 vs. S4) with minimal impact on *k*. However, the *Y*_M_ seems to decrease from 417 to 243 kPa. This variation indicates that connectivity and structural defects (e.g., weaker interparticle connection, heterogeneities, or microcracking) can impact the mechanical response, even when bulk density changes are small. For the 1.4 g series, TFA increased the stiffness under both EtOH conditions, although the accompanying trends in *ρ*_b_ and *k* depended on the non-solvent level. At EtOH = 0.8 mL, increasing TFA (S5 to S7) kept *ρ*_b_ nearly constant (0.233 to 0.240 g·cm^−3^) but increased the thermal conductivity from 42.5 to 50.5 mW·m^−1^·K^−1^ and raised the *Y*_M_ from 1222 to 2139 kPa, suggesting the formation of a more strongly connected skeleton that enhances both load transfer and solid conduction. At EtOH = 1.2 mL, increasing TFA (S6 to S8) reduced the *ρ*_b_ (0.274 to 0.230 g·cm^−3^) and *k* (49.4 to 45.5 mW·m^−1^·K^−1^) values while maintaining a high YM (approximately 2000 kPa), implying a microstructure that remained mechanically efficient at a lower solid fraction. In other words, improved connectivity and load-path efficiency can partially decouple stiffness from density and thermal conduction.

**Figure 2:**
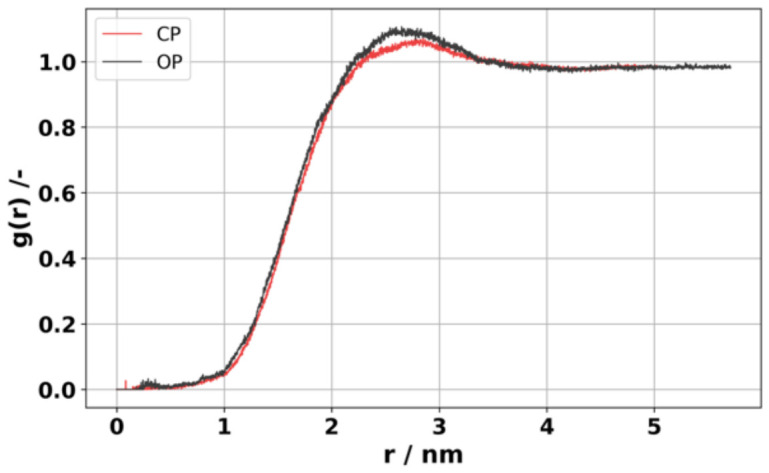
RDFs calculated between the COM of PET oligomers for the CP and OP systems.

Overall, these observations support the notion that TFA modulates the polymer solvation state prior to precipitation and shifts the demixing/arrest dynamics, thereby influencing the size of the structural features and the quality of interconnections within the network. Molecular Dynamics (MD) simulations were performed to deeply investigate these phenomena at the molecular level. The radial distribution function (RDF) is an effective tool for assessing the structural organisation of simulated systems. It characterises the spatial arrangement of particles by determining the probability of finding a particle at a given distance from a reference particle. The RDF profiles for the centre of mass (COM) of the PET oligomers, shown in Figure 2, characterised by broad, low-intensity peaks centred at approximately 2.8 nm instead of sharp, intense peaks, are consistent with a notable state of dispersion common to both systems (corresponding to the CP and OP formulations). This result suggests that both solvent mixtures behave as good solvents for rPET, promoting uniform dispersion, in which the local density of PET chains at long range closely matches the bulk density.

**Figure 3:**
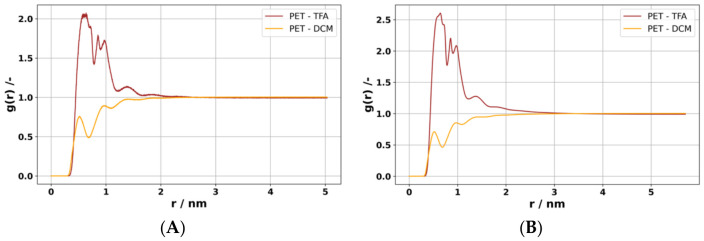
RDFs calculated between the COMs of PET monomers and TFA/DCM: (**A**) CP; (**B**) OP.

To further elucidate the solvation features of PET, the interactions between the polymer and each solvent mixture component were structurally and energetically analysed. The RDF results (Figure 3) reveal a clear preferential solvation of PET by TFA. The *g*(*r*) profile of PET-TFA exhibits a sharp, intense first peak at approximately 0.60 nm, indicating a significant local concentration of TFA molecules in the first solvation shell of the polymer. The presence of secondary and tertiary peaks further confirms that TFA induces a long-range structural order around the PET oligomers, likely driven by the strong electrostatic interactions and hydrogen bonding identified in the energy analysis (see below). In contrast, the RDF profile of PET-DCM exhibits a much weaker structural correlation; the first peak is notably less intense and slightly shifted toward larger distances. The fact that *g*(*r*) values for DCM remain below 1.0 at short distances indicates that DCM molecules are depleted in the immediate vicinity of the PET monomers compared to their bulk concentrations. This suggests that TFA molecules effectively displace DCM from the polymer surface, creating a TFA-rich protective layer that facilitates the dissolution process.

**Figure 4:**
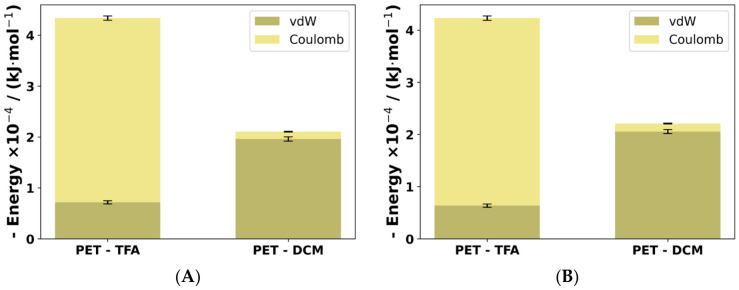
Interaction energies between PET and TFA/DCM: (**A**) CP; (**B**) OP. Error bars correspond to the standard deviation.

Figure 4 depicts the non-bonded interaction energy between PET and TFA/DCM, decomposed into Coulombic and van der Waals (vdW) contributions, with their respective error bars. The total interaction energy is significantly higher for the PET-TFA pair than for PET-DCM. This disparity primarily stems from the substantial difference in the Coulombic component. While PET-DCM interactions are almost exclusively governed by vdW forces, PET-TFA interactions are dominated by a strong electrostatic contribution. This high Coulombic energy is indicative of strong dipole–dipole interactions and, crucially, the formation of hydrogen bonds between the hydroxyl group of TFA and PET. In contrast, the PET-DCM interaction is markedly weaker and dominated by vdW forces. The negligible Coulombic contribution suggests that DCM does not participate in specific directional interactions with the polar functional groups of the polymer. Instead, DCM likely acts as a diluent in the mixture, helping to tune the viscosity and density of the medium without being the primary driver of solvation shell formation. The combination of the strong Coulombic energy and high-intensity RDF peaks for TFA confirms its role as the primary solvating agent, whereas DCM may act as a bulk medium component with minimal structural affinity for the polymer. This applies equally to both the central and optimal points.

**Figure 5:**
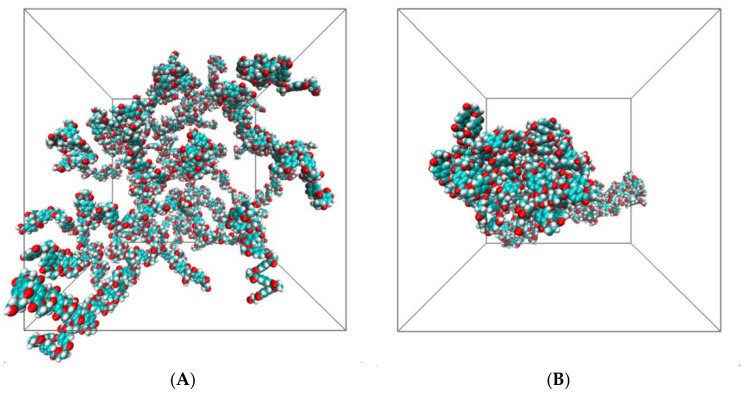
Final snapshot of the simulation box for system: (**A**) PET-TFA; (**B**) PET-DCM. The solvent molecules were omitted for better visualisation.

**Figure 6:**
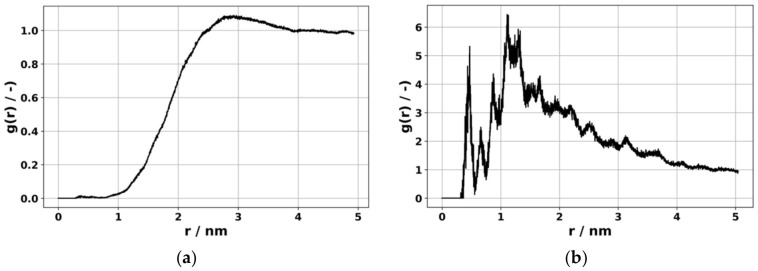
RDFs calculated between the COM of PET oligomers for binary systems: (**a**) PET-TFA; (**b**) PET-DCM.

Simulations of the binary systems PET-TFA and PET-DCM were performed to demonstrate the pivotal role of TFA in dissolving rPET. As clearly observed in Figure 5 (using CP as a representative case), the PET molecules are dispersed throughout the simulation box in the system with TFA. In contrast, the PET molecules are notably aggregated in the system containing DCM, indicating that DCM alone is insufficient to maintain the PET chains dispersed in solution, thereby demonstrating that TFA is essential for dissolving PET. This qualitative analysis is supported by the corresponding RDFs calculated between the COM of PET oligomers and respective solvent molecules (TFA or DCM; Figure 6). The RDF of the PET-TFA system is characterised by a broad peak that only slightly exceeds the bulk density at r = 3.0 nm. In contrast, the RDF of the PET-DCM counterpart reflects aggregation; several peaks are observed with *g*(*r*) values significantly greater than 1, indicating a strong preference for self-interaction over interaction with the solvent.

#### 2.1.2. Influence of the Non-Solvent

The amount of non-solvent (0.8 vs. 1.2 mL) controls the thermodynamic driving force for phase separation (precipitation) and can change the nucleation density, network coarsening, and extent of shrinkage during subsequent processing. Therefore, its effect strongly depends on the concurrent solvent affinity (TFA) and polymer concentration.

For the samples containing 0.7 g of rPET using 1.5 mL of TFA, increasing the concentration of EtOH led to a reduction in *ρ*_b_ from 0.151 to 0.108 g·cm^−3^ and a slight decrease in the thermal conductivity (40.7 to 39.2 mW·m^−1^·K^−1^). This behaviour is consistent with a more open structure and reduced solid conduction. When 2 mL of TFA was used, increasing the EtOH amount leads to a slight increase in *ρ*_b_, from 0.110 to 0.119 g·cm^−3^, with minimal change in *k*. However, in this case, the *Y*_M_ dropped to half of the value (524 to 243 kPa), again indicating that non-solvent-induced precipitation can produce microstructures of similar density but very different connectivity, with mechanical performance particularly sensitive to interparticle bonding and structural integrity. For the 1.4 g samples, with 1.15 mL of TFA, increasing EtOH led to a significant increase in all studied properties, suggesting that stronger precipitation produced a denser, more continuous network that improved both stiffness and heat conduction. However, when 2 mL of TFA was used, increasing the EtOH content slightly reduced the *ρ*_b_ (0.240 to 0.230 g·cm^−3^) and *k* values (50.5 to 45.5 mW·m^−1^·K^−1^) while maintaining the *Y*_M_ at high values. This combination indicates that under higher TFA, an increase in EtOH volume can yield a network with reduced solid conduction pathways without compromising load-bearing connectivity, reinforcing that the EtOH effect plays an important role in the structure formation.

**Figure 7:**
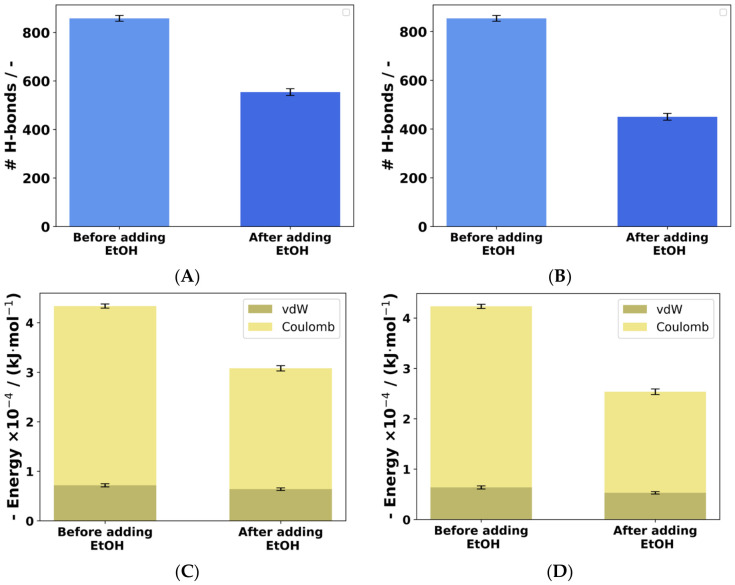
Number of H-bonds (TOP) and interaction energies (BOTTOM) between PET and TFA before and after adding EtOH: (**A**,**C**) CP; (**B**,**D**) OP. Error bars correspond to the standard deviation.

Like the role of the solvent in the properties of aerogels, the effect of adding a non-solvent has also been explored through MD studies. The effect of adding EtOH to the simulation boxes on the interaction between PET and TFA in both systems is summarised in Figure 7. As expected, there is a clear reduction in the number of H-bonds and a weakening of the interaction energies between PET and TFA after the addition of EtOH in both CP and OP. This trend reveals the role of EtOH as a precipitation agent by breaking the interactions between PET and TFA. However, this effect is moderate, as is desirable for the formation of a porous 3D network that spreads throughout the solution volume, and not the sudden precipitation of dense particles of the polymer.

### 2.2. Structural Properties

The microstructure of the rPET aerogel samples was analysed using the BET model. The surface areas and porosities of the rPET aerogels were evaluated for the CP and OP samples (Table 2).

The surface area of CP was almost twice that of OP. Despite the higher bulk density of the CP sample, the *k* value is similar to that of the OP sample. This can be attributed to the higher mesoporosity of the CP sample, which favours thermal insulation performance. The extensive mesoporous network and low amount of macropores can explain the low thermal conductivity obtained for these materials. In contrast, the lower SSA of the OP sample can be attributed to the lower amount of polymer material and the presence of more and larger macropores. This is in agreement with the differences observed between the values of pore size average in the micro- and mesopores range (BJH model) and averaged in all size ranges (last column of Table 2).

**Figure 8:**
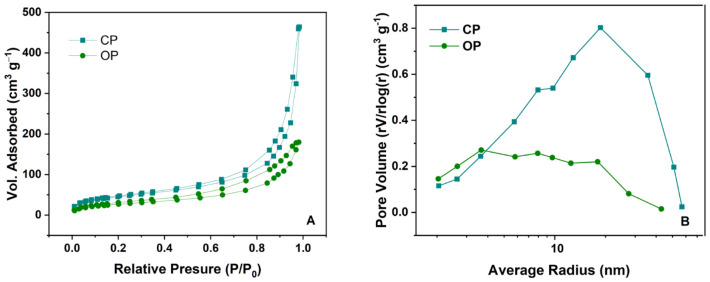
(**A**) N_2_ adsorption/desorption isotherms and (**B**) pore size distributions in the mesopore and small macropore regions for the CP and OP samples.

The isotherms and pore size distributions are shown in Figure 8. As can be observed, the CP and OP samples present very distinct structural features, which impact their properties, as explained before.

The typical adsorption–desorption isotherms (Figure 8A) for rPET aerogels correspond to type IV isotherms [16], but with a narrow hysteresis loop, suggesting a macropore-to-mesopore material. The *S*_BET_ values obtained here were similar to or lower than those found for other similar materials [6] (∼96 to 176 m^2^·g^−1^). The pore-size distribution obtained by the BJH model ranged from 1 to 60 nm, indicating an extensive mesoporous network, especially in the CP sample, although some micropores (diameter < 2 nm) were also detected. The curves continued in the range of pore sizes > 50 nm, indicating the presence of macropores in the samples. The increased mesoporosity of the CP sample may justify its higher *S*_BET_ value.

A lower density and specific surface area do not necessarily result in a lower thermal conductivity because heat transfer in these materials is governed by several simultaneous mechanisms. The total thermal conductivity of an aerogel generally has contributions from solid-phase conduction, gas-phase conduction, and radiative transfer [17]. Although a reduction in density typically decreases heat conduction through the solid skeleton, this reduction can be offset by an increase in other heat transfer contributions. At very low densities, the solid framework becomes increasingly sparse, reducing the attenuation of infrared radiation within the material. Consequently, radiative heat transfer can become more significant, partially compensating for the reduction in solid conduction. A lower surface area is often associated with coarser structures and larger pore diameters. In aerogels, the thermal insulation performance strongly depends on maintaining pore sizes below the mean free path of air molecules (i.e., below 60–70 nm) to promote the Knudsen effect, which suppresses gas-phase heat conduction. When the pore sizes increase (as in the case of the OP sample), gas molecules move more freely through the porous network, leading to a higher gas-phase thermal conductivity [17]. Consequently, an aerogel with a lower surface area may exhibit higher thermal conductivity despite its lower density.

**Figure 9:**
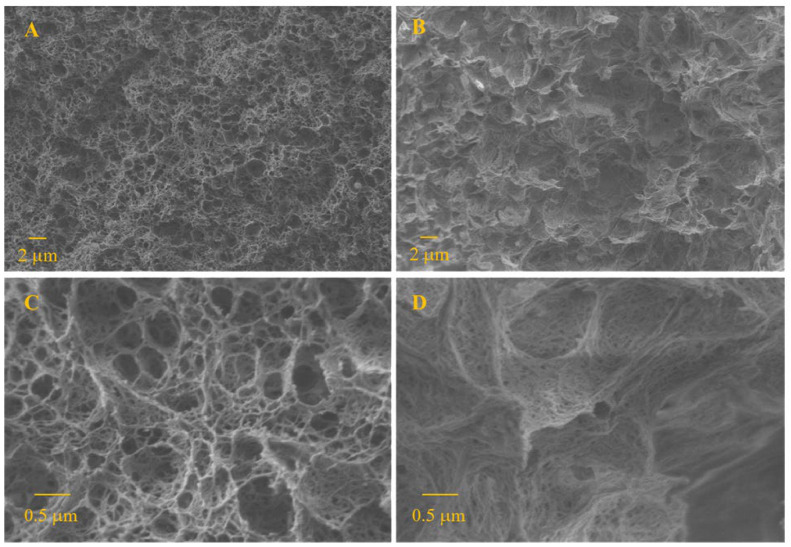
SEM images of (**A**,**C**) CP and (**B**,**D**) OP samples, at 2.5 and 20 k magnification, respectively.

SEM analysis was performed on the CP and OP samples to investigate the morphology of the rPET aerogels (Figure 9).

In general, both samples presented very different structures. The CP sample presented an apparently cohesive “lacy”-like structure with high porosity. The connectivity of the pores and the solid matrix is clearly visible, with pore sizes ranging from micro- to macropores, and the presence of mesopores is also observed. The OP sample exhibited numerous visible macropores (Figure 9D), which is consistent with the low density of this sample. This fragile structure, obtained with less TFA, led to a more deformed structure of the polymer matrix during the freeze-drying step (growth of ice crystals); moreover, the sample also deformed during the fracture before SEM analysis, contributing to the dragged polymer structure observed (Figure 9B).

**Figure 10:**
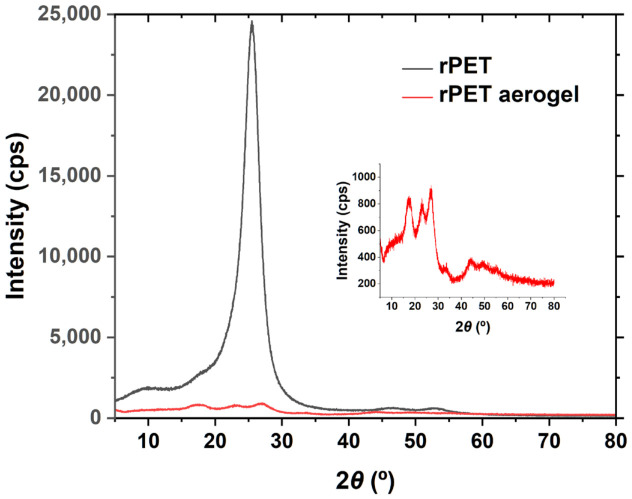
X-Ray diffractogram of the rPET from bottles and corresponding aerogel.

To evaluate changes in the material crystallinity during the process, X-ray diffraction was conducted on rPET before and after the dissolution/precipitation process (Figure 10). From the diffractograms, the rPET from bottles exhibits the typical semi-crystalline PET features, but with only one dominant reflection centred at 25.6°. This intense peak is commonly associated with the main PET crystalline peak, indexed in the literature to the (100) crystal plane [18]. This pattern indicates a strong preferential orientation towards the (100) plane, possibly due to PET processing. After processing into the rPET-based aerogel, the XRD pattern changes significantly, with three main crystalline peaks occurring at 17.5°, 22.9°, and 26.8°, corresponding to the (010), (110), and (100) crystal planes, respectively. In the aerogel, the crystalline pattern has peaks with much lower intensity, possibly due to reduced crystallinity and/or smaller crystalline domain size [19,20]. The lower diffraction intensity can indicate a higher amorphous fraction, i.e., reduced structural order of the aerogel sample [21]. The existence of all the peak positions in the aerogel material confirms a structure without preferential orientation, i.e., a more isotropic arrangement of the polymer chains, which is consistent with dissolution/precipitation processes.

Overall, the comparison indicates that the processing wet route used to produce the aerogel transforms the initially semi-crystalline rPET into a material with different crystallite sizes. This structural change is consistent with aerogel formation mechanisms where polymer chains are reorganised into a low-density network [22].

### 2.3. Thermomechanical Properties

The thermal conductivity values across all samples generally increased with *ρ*_b_, consistent with the increased contribution from solid-phase heat transport as the polymer skeleton became denser and more continuous [17]. This correlation demonstrates that the pore architecture and skeleton topology strongly influence the thermal transport phenomena. The CP sample exhibited a relatively high density (0.219 g·cm^−3^) and moderate stiffness (1089 kPa), yet a low *k* (39.0 mW·m^−1^·K^−1^), which is comparable to the lowest-density samples. This suggests that the CP composition promotes a porous network and skeleton architecture that limits effective heat transfer pathways while maintaining good mechanical integrity, having some enhanced influence of the Knudsen effect. This is also consistent with the global average pore diameter given in Table 2 (95.4 nm) which is not far from the mesopores upper limit, indicating that the sample has a significant amount of mesopores (see also Figure 8B), On the other hand, the presence of macropores limits the further decrease in thermal conductivity down to values featuring thermal superinsulation (<25 mW·m^−1^·K^−1^), typical of fully mesoporous silica aerogels.

**Figure 11:**
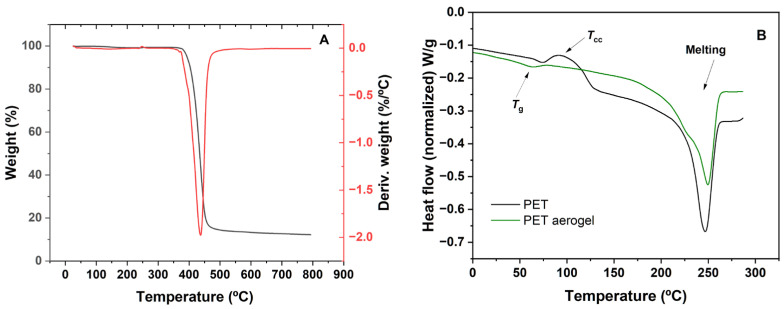
Thermal analysis by (**A**) TGA of rPET aerogel (OP sample) and (**B**) DSC of rPET aerogel (OP sample) and used PET. Endothermic events are shown as downward peaks, while exothermic events are upward peaks.

Figure 11 shows the TG/DTG and modulated DSC profiles of the rPET aerogel measured in a nitrogen atmosphere. The TG curve remains constant up to approximately 380−400 °C, indicating the good thermal stability of the rPET aerogel. A single dominant mass loss event occurs between 400 and 500 °C (DTG peak at ~438 °C). This behaviour is characteristic of the primary pyrolytic decomposition of PET, which is dominated by random chain scission and volatilization of oligomeric/aromatic degradation products [23,24]. Above 500 °C, only minor additional mass loss is observed, yielding a residual mass of ~15 wt% at 800 °C. The presence of this residue under N_2_ suggests the formation and stabilisation of a carbonaceous char, which is expected for aromatic polyesters and can be promoted by the aerogel morphology, favouring carbonization and retention of solid products [25].

The modulated DSC data (Figure 11B) exhibit well-defined transitions commonly reported for semi-crystalline PET [26], where three key events were observed. The glass transition of PET (*T*_g_) occurs at approximately 75 °C, which is consistent with the *T*_g_ of PET, typically occurring at approximately 75–80 °C. In the case of the PET aerogel sample, the *T*_g_ appears at 62 °C. The lower *T*_g_ of the PET aerogel suggests higher chain mobility, likely arising from its porous network and increased free volume. The second event, at approximately 90 °C, is characterised by a broad, weak exothermic peak, compatible with a cold crystallisation (*T*_cc_). The presence of this peak in the PET sample indicates that its crystalline domains were not fully developed during prior processing, allowing further crystallisation upon heating. This event is absent in the aerogel sample, suggesting that crystallisation was completed during its processing. The third event, at approximately 250 °C, corresponds to the melting and is observed both in the PET sample and in the PET aerogel. However, the melting peak in the aerogel is broader than in the PET sample, indicating a wider distribution of crystal sizes and/or less uniform crystalline domains within the aerogel structure.

**Figure 12:**
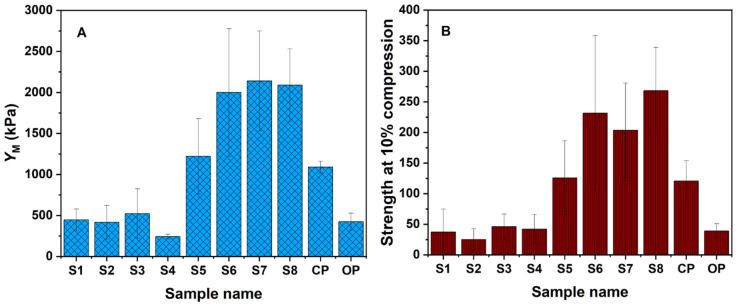
Mechanical performance of rPET aerogels. (**A**) Young’s modulus and (**B**) strength at 10% compression.

The mechanical performance of the samples was investigated using a compression/decompression test up to a 15% strain. The obtained mechanical properties (Figure 12) showed a stronger sensitivity to the composition-driven microstructure than to the thermal conductivity. Samples with similar *ρ*_b_ displayed markedly different *Y*_M_ (e.g., S3 vs. S4), highlighting that *Y*_M_ depends not only on the solid fraction but also on the defect density and load-path efficiency of the network produced during phase separation (precipitation) [27].

The Young’s modulus and compressive stresses at 10% strain are important indicative parameters of the mechanical stiffness of the materials and their behaviour under compressive forces, which may be important to assess their ability to be easily handled or/and used in applications that need to support some load level, e.g., in floors, walls, or rooftops.

The strength is taken at 10% compressive stress, according to the standards ISO 844:2021 (ISO, 2021) [28] and ISO 22482 (ISO, 2021) [29], which, in this case, is in the elastic region of the deformation curve. Thus, the obtained values (Figure 12B) are in accordance with the Young’s modulus trend varying from 25 to 268 kPa, with the samples containing 1.4 g of polymer presenting a higher compressive strength between 125 and 268 kPa. According to the ISO 22482 standard, the rPET aerogels in this study correspond to the CS(10/Y)25 to CS(10/Y)100 levels. Considering that this standard specifies a minimum level of CS(10/Y)1, these levels place our material at a higher limit.

Compared to other PET-based aerogels reported in the literature, the aerogels reported in this study have a higher Young’s modulus [7,12]—this result is expected, as the other designated PET aerogels are predominantly a mat of entangled PET fibres with inherent flexibility.

Regarding the behaviour under compression–decompression loads, after a 15% strain, the PET aerogels usually only recover up to 35% of the initial sample dimensions (Figure S1 of Supplementary Information). This fact is associated with the already mentioned stiffness of the samples and the development of some structural defects during the compression phase of the test. Reinforcement strategies with fibres can be considered to improve the mechanical flexibility of the material if required for the application.

## 3. Conclusions

In this study, rPET-based aerogels were prepared using a room-temperature dissolution/precipitation method. The absence of high processing temperatures reduces energy consumption during manufacturing, potentially lowering the overall carbon footprint of the material. Moreover, when derived from recycled PET waste, these aerogels contribute to plastic waste valorisation and support circular economy strategies by transforming discarded materials into high-value environmental products.

The influence of different preparation parameters was investigated and optimised using DoE to systematically evaluate the input preparation factors and their effects on the output responses, thereby optimising the process. Increasing the rPET content shifted the aerogels toward higher density, thermal conductivity, and significantly higher stiffness. In contrast, tuning the TFA and EtOH allowed for adjusting the phase-separation pathway and network connectivity, enabling variations in *Y*_M_. The highest stiffness was achieved for the 1.4 g rPET-containing formulations (up to ~2.1 MPa), while the lowest thermal conductivities (~39 mW·m^−1^·K^−1^) were obtained in low-density samples and in the case of the CP sample, indicating that solvent/non-solvent balance can be used to obtain low thermal conductivity values, even at moderate densities.

The obtained results compare satisfactorily with other materials reported in the literature (Table 3).

These results demonstrate that rPET aerogel properties can be systematically tailored by combining the polymer content with targeted solvent quality and non-solvent adjustments, tuning the microstructure and connectivity in a thermodynamically governed dissolution/precipitation process.

These findings were supported by MD simulations, which showed that a minimum amount of TFA is needed to achieve good dissolution and that the amount of non-solvent also affects the precipitation process by moderately reducing the solvent affinity.

The developed low-energy process can be used as an upcycling strategy to recycle PET and obtain value-added products for thermal insulation or other surface/porosity-dependent applications.

## 4. Materials and Methods

### 4.1. Materials

Recycled poly(ethylene terephthalate) (rPET) was obtained from waste PET bottles that had been completely or partially recycled. Dichloromethane (DCM; 99.95%) and ethanol (EtOH; 96%) were obtained from Fisher Scientific (Porto Salvo, Portugal). Trifluoroacetic acid (TFA; >99.0%) was obtained from TCI Europe (Zwijndrecht, Belgium). Ultra-pure water was locally obtained by reverse osmosis using Diwer Technologies equipment. All chemicals were used as received, without additional purification steps.

### 4.2. Preparation of rPET Aerogels

**Figure 13:**
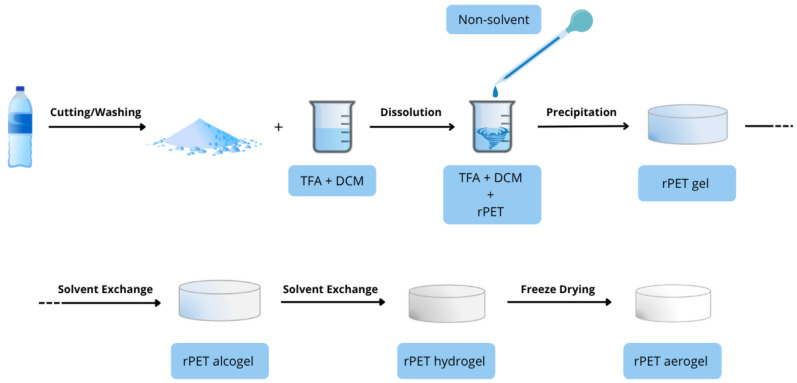
Schematic representation of the rPET aerogel sample preparation procedure.

The rPET-based aerogels were prepared at room temperature using a dissolution/precipitation method. First, the rPET bottles were thoroughly washed with ethanol and water to remove any impurities and dried overnight at 60 °C. The samples were then cut into small pieces. Different amounts of polymer (0.7–1.4 g) were placed in a DCM:TFA co-solvent mixture with different co-solvent ratios (Table 1) and magnetically stirred for 15 min to completely dissolve rPET. During dissolution, solvent molecules (TFA/DCM mixture) diffuse into the amorphous regions, increasing the polymer chain mobility, and consequently, the crystalline regions progressively loosen and disperse uniformly. A transparent and clean solution was obtained. After dissolution, precipitation was induced by the slow addition of the non-solvent (ethanol), which decreases the solvent quality and rPET solubility, leading to chain aggregation through intermolecular interactions. Magnetic stirring was continued until the viscosity of the solution began to increase. The gel was typically formed within a few minutes. The process for rPET aerogel preparation is illustrated in Figure 13.

After an overnight ageing period at 27 °C, the gels were washed three times with EtOH (12 h/wash), and three additional washes with distilled water (12 h/wash) were performed to replace the EtOH in the porous network. Finally, the hydrogels were freeze-dried for 48 h. Prior to freeze-drying, the gel was pre-frozen for 1 h in the refrigerated chamber of the freeze-dryer at −80 °C.

### 4.3. Morphological and Structural Properties

The bulk density (*ρ*_b_) values of the samples were assessed by measuring the weight in a 10^−5^ g precision microbalance and the respective volume of the samples. The specific surface area (*S*_BET_) and pore size were estimated by nitrogen gas adsorption at 77 K by applying the Brunauer–Emmett–Teller (BET) and Barrett–Joyner–Halenda (BJH) theories to the adsorption and desorption branches of the isotherm, respectively, using an Autosorb iQ from Quantachrome Instruments (Anton-Paar Group, Graz, Austria).

The microstructure and morphology of the rPET aerogel samples were observed by scanning electron microscopy (SEM) using a Compact/VP Compact FESEM (Zeiss Merlin) (Carl-Zeiss, Jena, Germany) after coating the samples with a thin gold layer for 45 s. The working distance was approximately 4.5–6 mm, and the voltage was 1 kV.

The crystalline structures of raw and processed rPET were evaluated using X-ray diffraction (XRD). XRD patterns were obtained using a Rigaku SmartLab SE diffractometer operating in Bragg–Brentano geometry (θ–2θ) and CuKα radiation (λ = 0.15418 nm). The information was collected in the range of 5–80° (2θ), with a step size of 0.02°, at 295 K. The phases were identified using SmartLab Studio II software.

### 4.4. Thermomechanical Characterisation

The thermal conductivity, *k*, was measured at 20 °C applying the transient plane source method in a Thermal Constants Analyser model TPS 2500 S from Hot Disk^®^ (Hot Disk AB, Gothenburg, Sweden). A 5501 sensor (diameter = 6.4 mm) was applied according to the dimensions of the samples, and two replicates of the samples were used for each measurement to sandwich the sensor. Two independent measurements were performed for each sample. The thermal stability of PET-based aerogels was assessed using a Simultaneous Differential Thermal Analysis (SDT), TA Instruments TGA Q600 (TA Instruments, New Castle, DE, USA), from room temperature to 600 °C, with a heating rate of 10 °C·min^−1^. The Modular Differential Scanning Calorimetry (MDSC) was performed on a TA Instruments Discovery Series DSC25 (TA Instruments, New Castle, DE, USA) with a heating rate of 10 °C·min^−1^. All experiments were conducted under an N_2_ atmosphere.

The mechanical properties were assessed by uniaxial compression–decompression tests in an Inspekt mini-series (Hegewald and Peschke), using a 3 kN load cell at a rate of 1 mm.min^−1^. The Young’s modulus (*Y*_M_) and elasticity/flexibility of the samples were assessed by performing compression and decompression tests (up to 15% compressive strain). The compression test was conducted following the ASTM standard D695-02a (ASTM, 2002) [31], and the Young’s moduli were obtained from the elastic region of the stress–strain curves. Moreover, the compressive stresses at 10% strain were evaluated to assess the mechanical resistance of the samples according to the standards ISO 844:2021 and ISO 22482 [28,29].

### 4.5. DoE Analysis

For the DoE analysis, three synthesis parameters (factors) were selected: (1) polymer concentration (rPET), (2) co-solvent amount (TFA), and (3) non-solvent amount (EtOH). A full factorial (2^3^ runs) with two levels for each factor (S samples in Table 4) and one central point (CP in Table 4) was evaluated. The levels of the factors (Table 1) were established based on preliminary results [6] and additional exploratory experiments. The DCM amount was always established to have a total volume of TFA + DCM of 7 mL, so it was not considered as an independent factor.

The bulk density, thermal conductivity, and Young’s modulus were selected as the responses for the DoE screening. The influence of the main parameters on these properties, as well as their first-order interactions, was evaluated using the standard least-squares fit model. To calculate the optimum point (OP) of the system, the desirability function was used to determine the best synthesis conditions. The weights considered were 0.5, 0.3, and 0.2 for the thermal conductivity, bulk density, and Young’s modulus, respectively. All DoE tests were performed using JMP (version 19) software licensed to the University of Coimbra.

### 4.6. Molecular Simulations

Molecular dynamics (MD) simulations of the CP and OP formulations were performed to provide insights into the dissolution and precipitation of rPET. Additional simulations including EtOH in both systems were carried out to evaluate its impact, alongside simulations of binary PET/TFA and PET/DCM mixtures to demonstrate the critical role of TFA.

All simulations were performed using GROMACS 2025.3 [32]. The CHARMM General Force Field was used for all components of the system [33,34]. While using full-length polymer chains would more closely mimic the experimental conditions, it would make the simulations impractical. Therefore, PET was represented by oligomers of 10 repeating units (DP = 10) [35,36] to balance the representativeness and computational efficiency (it is worthwhile to mention that running a simulation with 20 units under the central-point conditions led to no significant changes in the results). The molecules were randomly placed in a cubic simulation box with a side length of 15 nm. This box size was selected considering the PET molecular length (~11 nm), ensuring compliance with the minimum image convention. The number of molecules was calculated to reflect experimental concentrations, with the volume of each solvent corresponding to 7 mL for the binary PET/TFA and PET/DCM mixtures. These quantities are listed in Table 5.

All simulations were carried out under ambient conditions, viz. 293 K and 1 bar. Production runs were performed for 500 ns under periodic boundary conditions. Further simulation details on models, parameters and conditions are provided in the Supplementary Information, supported by the References [37,38,39,40].

## Figures and Tables

**Table 1:** Parameters used in the preparation of rPET aerogels and the obtained main properties used for the design of experiments (DoE) analysis.

Sample	rPET (g)	DCM (mL)	TFA (mL)	EtOH (mL)	*k* (mW·m^−1^·K^−1^)	*ρ*_b_ (g·cm^−3^)	*Y*_M_ (kPa)
S1	0.7	5.85	1.15	0.8	40.7 ± 1.3	0.151 ± 0.030	446 ± 134
S2	0.7	5.85	1.15	1.2	39.2 ± 2.7	0.108 ± 0.001	417 ± 206
S3	0.7	5.0	2.0	0.8	39.4 ± 1.0	0.110 ± 0.008	524 ± 300
S4	0.7	5.0	2.0	1.2	40.1 ± 1.3	0.119 ± 0.012	243 ± 28
S5	1.4	5.85	1.15	0.8	42.5 ± 0.9	0.233 ± 0.009	1222 ± 460
S6	1.4	5.85	1.15	1.2	49.4 ± 4.7	0.274 ± 0.045	2000 ± 777
S7	1.4	5.0	2.0	0.8	50.5 ± 0.6	0.240 ± 0.032	2139 ± 610
S8	1.4	5.0	2.0	1.2	45.5 ± 3.7	0.230 ± 0.017	2090 ± 442
CP	1.05	5.42	1.58	1.0	39.0 ± 2.4	0.219 ± 0.033	1089 ± 72

**Table 2:** Microstructural properties of the CP and OP samples.

Sample	SSA (*S*_BET_) (m^2^·g^−1^)	BJH Pore Volume (^a^) (*V*_p,BJH_) (cm^3^·g^−1^)	BJH Average Pore Size (^a^) (nm)	Total Pore Volume (^b^) (*V*_p,T_) (cm^3^·g^−1^)	Average Pore Diameter (^b^) (nm)
CP	151.5 ± 1.9	0.82 ± 0.18	11.6 ± 0.1	3.6	95.4
OP	85.2 ± 4.0	0.25 ± 0.02	6.2 ± 1.4	8.2	386

^a^ The pore size distribution and pore volume were calculated using the desorption branch of the Barrett-Joyner-Halenda (BJH) model. ^b^ Total pore volume and average pore diameter calculated according to Equations (2)–(4) in Reference [6].

**Table 3:** Overview of rPET-based porous materials for thermal insulation.

PET Processing Method	Final Material	Thermal Conductivity (W·m^–1^·K^–1^)	Density (g·cm^−3^)	Ref.
Cross-linked fibre-based materials:				
rPET fibres	PET, crosslinkers PVA and GA	0.035–0.038	0.007–0.026	[7]
rPET fibres	PET, crosslinkers PVA and GA	0.0318–0.0348	0.014–0.062	[12]
rPET fibres and bonded lattice	PET, crosslinker PVA	0.033–0.038	0.042–0.072	[30]
rPET fibres	PET, Fly Ash, crosslinkers PVA and Xanthan Gum	0.035–0.040	0.045–0.060	[8]
Continuous matrix-based materials:				
Dissolution–precipitation	PET aerogel with PS and quartz fibre reinforcement.	0.0392–0.0474	0.15–0.21	[6]
Dissolution–precipitation	PET aerogel	0.038–0.047	0.11–0.28	Present work

**Table 4:** Factor levels used in the design of experiments (DoE) for the preparation of rPET aerogels.

Sample	rPET	DCM	TFA	EtOH
**S1**	-	+	-	-
**S2**	-	+	-	+
**S3**	-	-	+	-
**S4**	-	-	+	+
**S5**	+	+	-	-
**S6**	+	+	-	+
**S7**	+	-	+	-
**S8**	+	-	+	+
**CP**	000	000	000	000

**Table 5:** Composition of the simulation box.

	Number of Molecules					
System	PET	TFA	DCM	EtOH	Total	Total Number of Atoms
PET/TFA	42	7051	-	-	7093	65,774
PET/DCM	42	-	8433	-	8475	51,531
CP	42	1591	6530	-	8163	54,744
OP	42	1748	10,635	-	12,425	76,525
CP w/EtOH	42	1591	6530	1321	9484	66,633
OP w/EtOH	42	1748	10,635	2392	14,817	98,053

## Data Availability

The data presented in this study are available from the corresponding authors upon reasonable request.

## Supplementary Materials

The following supporting information can be downloaded at https://www.mdpi.com/article/10.3390/gels12060521/s1. Molecular dynamics parameters and conditions details and Figure S1: Stress-strain curves obtained by the compression-decompression test up to 15% strain for the CP and OP samples.
