# Supplementary material for: Optimising Fully rPET-Sourced Aerogel Production Using a Sustainable Dissolution–Precipitation Approach
Source: Gels. 2026 Jun 10;12(6):521. doi: 10.3390/gels12060521 (PMC13298159; doi:10.3390/gels12060521)
Supplement: Supplementary file 1 [file gels-12-00521-s001.zip › gels-4321946-supplementary.pdf]

Supplementary Materials

on

Optimising Fully rPET-Sourced Aerogel Production Using a Sustainable Dissolution–Precipitation Approach

Cláudio M. R. Almeida[1], David Gonçalves[1], Brigite Jorge[1], Pedro C. F. Silva[1], Tiago Cardoso[1], Pedro Nuno Simões[1], Ana C. Fonseca[2*], Luisa Durães[1*]

[1]University of Coimbra, CERES, Department of Chemical Engineering, 3030-790 Coimbra, Portugal
[2]University of Coimbra, CEMMPRE, ARISE, Department of Chemical Engineering, 3030-790 Coimbra, Portugal

Keywords: Polymer waste upcycling, rPET dissolution, Polymer aerogels, Thermal insulation

[*]**Corresponding authors:** Cláudio Almeida (claudio@eq.uc.pt); Luisa Durães (luisa@eq.uc.pt); Ana C. Fonseca (anafs@eq.uc.pt)

Molecular dynamics parameters and conditions

The initial structures were generated using CHARMM-GUI [37], which provided the coordinates, topology, and parameter files compatible with GROMACS. PET files were generated using the Polymer Builder tool [38], whereas all other molecule files were generated using the Ligand Reader & Modeler tool [39]. The system setup was generated with GROMACS tools.

The systems were first energy-minimized using the steepest descent algorithm to remove unfavourable contacts. A maximum force tolerance of 250 kJ/mol.nm was applied. Following minimization, the systems were equilibrated under the NVT (constant number of particles, volume, and temperature) and NPT (constant number of particles, pressure, and temperature) ensembles, each for 1 and 5 ns, respectively, using a V-rescale thermostat at room temperature (293 K) and a C-rescale barostat at 1 bar. Production runs were performed for 500 ns under periodic boundary conditions.

A time step of 2 fs was used, and long-range electrostatics were treated using the Particle Mesh Ewald (PME) method. Van der Waals interactions were calculated using a 1.2 nm cutoff. All bonds involving hydrogen atoms were constrained using the LINCS algorithm to allow for the selected integration time step.

All analyses were performed using the built-in GROMACS tools and Python. The trajectories and structural snapshots were visualized using the Visual Molecular Dynamics (VMD) software [40].

Mechanical compression/decompression performance of the rPET aerogel

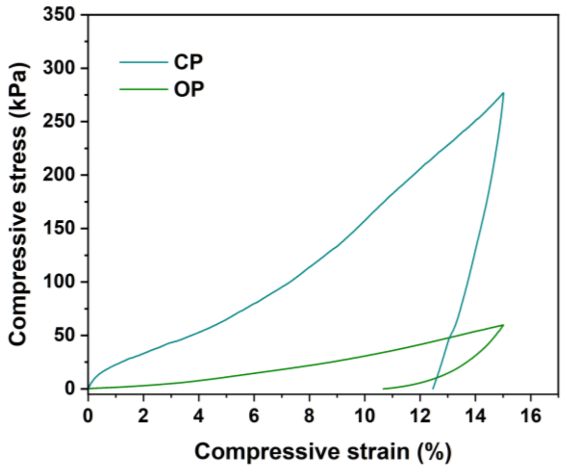

Figure S1. Stress-strain curves obtained by the compression-decompression test up to 15% strain for the CP and OP samples.
